# Biomedical, socio-behavioral, and implementation science gaps in multipurpose prevention technology research

**DOI:** 10.3389/frph.2023.1244659

**Published:** 2023-09-01

**Authors:** James E. Cummins, C. Leigh Allen, Sonia Lee, Theresa E. Senn

**Affiliations:** ^1^ Preclinical Microbicide and Prevention Research Branch (PMPRB), National Institute of Allergy and Infectious Diseases (NIAID), Rockville, MD, United States; ^2^ Contraception Research Branch (CRB), Eunice Kennedy Shriver National Institute of Child Health and Human Development (NICHD), Bethesda, MD, United States; ^3^ Maternal and Pediatric Infectious Disease Branch (MPIDB), Eunice Kennedy Shriver National Institute of Child Health and Human Development (NICHD), Bethesda, MD, United States; ^4^ HIV Prevention and Care Continuum, Co-Morbidities, and Translational Research Branch, National Institute of Mental Health (NIMH), Rockville, MD, United States

**Keywords:** multipurpose prevention technology, sexually transmitted infections, contraception, socio-behavioral, drug co-formulation, regulatory affairs, end-user preferences, implementation science

## Abstract

There is strong global need for the development of Multipurpose Prevention Technologies (MPTs) that prevent HIV, pregnancy, and/or other sexually transmitted infections (STIs). However, despite decades of research focused on the development of MPTs, numerous research gaps remain, contributing to reproductive health disparities. This commentary will highlight biomedical, socio-behavioral, and implementation science gaps in MPT research. Biomedical gaps and barriers include limited dosage forms, challenges around drug selection and stable coformulation of multiple drugs, and an unclear regulatory pathway. Behavioral, social, and structural gaps include lack of research around MPT preferences for some subgroups of potential end users, lack of knowledge around whether MPTs improve uptake, adherence, and persistence vs. separate products, and a need to further understand how social and cultural factors might impact MPT interest and use. Gaps in implementation science research will need to be addressed to better understand how to implement MPTs to maximize effectiveness and benefit. This commentary will also identify opportunities for integrating biomedical and behavioral science around MPTs.

## Introduction

Globally, increasing reproductive health options for women will address health disparities and further opportunities to address gender inequality. There is an unmet need for contraception among women, and concurrently, an unmet need for prevention of HIV and/or other sexually transmitted infections (STIs) among women. The HIV prevention landscape has been transformed with FDA approval of Truvada (TDF/FTC) as oral PrEP in 2012, Apretude (CAB-LA) as long-acting injectable PrEP in 2021, and more recently with approval of the dapivirine intravaginal ring in several countries in Eastern and Southern Africa. Indeed, the development of Multipurpose Prevention Technologies (MPTs) to prevent pregnancy, HIV, and/or other STIs is an opportunity for collaborative and innovative efforts among product developers, regulatory bodies, biomedical researchers, behavioral and implementation scientists, and most importantly, end users. Although advancements in both contraception and HIV/STI prevention methods offer potential platforms for MPTs, significant barriers persist that impact their real-world effectiveness. In this commentary, current advances in MPTs are briefly summarized and challenges, gaps and further research directions are identified.

## Biomedical gaps and development challenges for MPTs

### Selection of drugs and dosage forms, co-formulation of multiple drugs, and product scale-up/manufacture

The pace of MPT development has trailed that of non-vaccine biomedical HIV prevention with biomedical gaps and development challenges that include a limited number of drug choices, concerns around drug potency and drug loads that impact long-acting formulations, the physiochemical compatibility of co-formulating multiple drugs, the potential for drug-drug interactions, and challenges around scale-up and manufacture of novel dosage forms (i.e., formulation delivery platforms). In fact, these challenges have impacted development of MPTs with activity against HIV or non-HIV STIs, in combination with contraception, since multiple drugs are required. As a result, there are fewer innovative dosage forms in late clinical testing (oral pills and vaginal gels) compared to more innovative dosage forms in early clinical testing (intravaginal rings and fast dissolving inserts) or preclinical development (vaginal films, implants, and microarray patches).

Approved drugs are typically used to expedite the drug development process and rapidly advance a formulation into early clinical testing. A majority of MPT products in development contain licensed or approved antiretroviral drugs (ARVs) for HIV in combination with licensed contraceptives (1, 2). However, there are MPTs in development for non-HIV STIs that contain antivirals specific for HSV (TFV, TAF, and acyclovir) or broad-spectrum agents (Yaso-Gel, VivaGel, and Q-Griffithsin) with activity against both bacterial (gonorrhea and chlamydia) and viral (HPV and HSV) pathogens (2). The two most advanced products in clinical testing are Dual Prevention Pills (DPPs): a daily oral capsule containing two pills, TDF/FTC pill + LNG/EE pill (3), and a daily bilayer oral tablet containing TDF/FTC and LNG/EE (4). Despite their potential for rapidly taking an MPT to market, these DPPs may not represent the ideal MPT given their large size and possible issues around adherence to a daily capsule or tablet.

Several novel MPT dosage forms in development have their own unique challenges. More potent drugs are often required for long-acting formulations (e.g., IVRs, implants, and patches) to ensure drug loads that will deliver sustained concentrations over longer periods of times (i.e., weeks to months or longer) with minimal delivery volumes (5). Most current drugs are not potent enough to ensure minimal volumes and sizes that are acceptable to end users. As a result, product developers are now using prodrug chemical modifications (e.g., drug-polymer conjugates) to modulate physiochemical and pharmacokinetic (PK) properties or novel drug delivery platforms (e.g., biodegradable hydrogel depots) as approaches to address drug potency and control extended drug release (5, 6). It remains to be determined whether these approaches will result in viable next generation MPTs that deliver multiple drugs over longer periods.

While diverse preferences have driven a range of contraceptive types, dissatisfaction with current contraceptives indicates that there is considerable room for new and improved products (7). There is a need for more discreet options with fewer sides effects, including non-hormonal options and long-acting formulations (7). There will likely be a more complex regulatory pathway for new drugs or alternative contraceptive technologies to be considered for MPTs when these are used in combination with other licensed drugs.

It can be difficult to combine multiple drugs into a single product formulation that can deliver stable, sustained release of each drug and maintain the PK/therapeutic targets over extended periods – particularly when drugs have different physiochemical properties. While *in vitro* systems and animal studies are often used to assess and evaluate prototype formulations, there are no universally accepted protocols for evaluating novel drug delivery platforms in *in vitro* release studies (8) or relevant animal models, particularly those that assess vaginal products (9). The potential for drug-drug interactions should not be underestimated – particularly when certain drugs are known to induce metabolizing enzymes that could result in increased hormone metabolism – thus possibly impacting contraceptive effectiveness (8). MPT developers will need to consider multiple approaches to address these challenges and generate preclinical data that is acceptable to regulatory authorities and informative for early clinical testing.

As product developers focus on more novel drug delivery platforms (next generation IVRs, implants, and microarray patches), there may be challenges around transfer and scale-up of these MPT products due to limitations in current manufacturing processes. Technological considerations should be considered early in the drug development pathway to ensure viable end products by identifying and overcoming potential downstream issues related to material choices, drug compatibility, drug loading, and controlled release parameters (8).

### Unclear US regulatory pathway

The MPT community has benefitted from increased communication between funding bodies, multidisciplinary conversations between basic, clinical and socio-behavioral scientists, and increased collaborative engagement from the FDA to clarify regulatory requirements (10). Despite improvements, a clear path to FDA approval remains elusive and, in looking at the global landscape, many of the same challenges are likely to impact regulatory approval of MPTs outside the US. Young Holt et al. offer a primer for regulatory considerations amidst a strategic path forward for the field (11, 12), and Hemmerling et al. outline many of the unique regulatory hurdles faced by MPT developers (10). Although the FDA has drafted new bioequivalence guidance, knowledge gaps remain, including whether bioequivalence will be a suitable surrogate for efficacy studies.

Ethical quandaries can arise when working with vulnerable populations and testing experimental contraceptives. For example, effective contraceptive use is often a requirement of HIV trials, yet cessation of contraception is needed to assess the contraceptive indication of a new combination product (13). Combining two unrelated indications into a single trial requires a design that may be prohibitively complicated. Thus, some developers may choose to seek approval for a single indication while gathering exploratory data and then repeat the approval process for an additional indication, but there are drawbacks to this approach (increased time and money).

Another gap lies between the discovery of new products and translating them into a clinical success. While an IND/IDE represents an early milestone in the product development journey, it is never too early to consider the target market, ideal product attributes, and engage experts in a regulatory strategy. The potential high profits of the US market are often the focus of product development yet, for MPTs, early efforts to include a strategy for introducing a product to the global market will be beneficial, including a consideration for applying to the WHO Prequalification of Medicines Programme (PQP)^1^. Increased collaboration is key for the success of MPTs, as they have the potential to benefit countless users worldwide.

Several briefs authored over a decade ago are still relevant today for MPT developers seeking regulatory perspectives. Romano et al. (14) propose scenarios based on FDA guidance that illustrate nonclinical testing needs, regulatory considerations, and routes toward approval. While the FDA has since issued clarifying documents for combination products (defined by 21 CFR 3.2e^2^), many principles discussed remain true, including the reminder that the journey for each pharmaceutical product will be different. Concrete answers to generalized questions on MPT guidance are likely unobtainable, so adaptability to a changing landscape is key. The road map at the outset will not be what is in the rearview mirror at the end. Brady and Park (15) provided a guide to key regulatory documents from FDA, EMA and ICH, which are still relevant even though guidance such as Principles of Premarket Pathways for Combination Products^3^ (FDA) and Clinical Development of Fixed Combination Medicinal Products^4^ (EMA) have been added.

Brady (16) noted that the uncertainty of the regulatory environment for MPTs disincentivizes investment and thus advancement, but the potential impact of these products is too important to forgo. From 2018 to 2022, biomedical research funding provided by the NIH eclipsed any other funder for HIV, HPV, and other STIs (17). During this same period, the NIH remained one of the top two funders for research on contraception and development of contraceptive-containing MPTs (17). However, even with this investment, the NIH alone is not equipped to usher products from discovery to market. With the vast array of products in the current pipeline (2) and the aforementioned underinvestment in this area, public-private partnerships and other funding sources are imperative for MPTs to succeed.

FDA decisions and guidance, such as primary mode of action (PMOA) for combination products, may be simplified if a product exists for comparison, but until then primary jurisdiction for premarket review and regulation of a combination product will be assigned based on an algorithm (21 CFR 3.4^5^) (18). Once several different MPTs are established, MPT advancement will be enabled by the path elucidated by these trailblazing products. Until then, the FDA urges developers to have conversations via request for designation (RFD) or pre-RFD avenues. In fact, a unifying theme for the information available about MPT regulatory matters and combination products is communication, including but not limited to requests for clarification and conversations to fill knowledge gaps with regulatory agencies as soon as possible during development and often thereafter. Hopefully, developers will continue to communicate lessons learned while navigating the regulatory environment (see: MPT Regulatory Pathways: Case Studies from MPT Product Developers^6^), so the long sought-after MPT regulatory road map can be created collectively.

## Socio-behavioral gaps

### Preferred MPT characteristics

Both women and men are highly interested in novel MPTs and indicate they would prefer a combined product over separate methods for the prevention of HIV, STIs, and/or pregnancy (19, 20, 21). The only MPTs currently available are male and female condoms, which protect against pregnancy, HIV, and other STIs. However, there are numerous barriers to condom use, including concerns over loss of pleasure, stigma, and concern that condoms signal a lack of trust in the relationship; additional barriers to use of the female condom include concerns about the size, lack of partner acceptance, and difficulty with insertion (22, 23, 24). Despite the ease of a single medication for both purposes, fertility desires and perception of HIV/STI risk may change over time, and users may no longer desire one of the MPT indications. Research is needed to understand how preferences for MPT use change over time, and how switching from an MPT to a single indication product (and vice versa) can be supported.

Behavioral science research has begun to identify the preferred characteristics of an MPT, which can help guide MPT developers in the types of products in which they invest development resources. There are three main lines of research from which we have learned about product preferences: qualitative interviews, often conducted after participation in early stage MPT trials, discrete choice surveys (a survey in which participants are presented with a pair of products that differ in their attributes and asked to choose which product they prefer, across multiple product pairs), and placebo studies.

Qualitative studies have found that factors such as discreetness, reversibility, longer duration of protection, and community acceptance are important characteristics of MPTs (25, 26, 27, 28). A discrete choice survey of couples conducted in Uganda and Zimbabwe investigated couple preferences for an MPT, as well as how individual preferences for an MPT differ from couple preferences. This study found that the combination of product form and dosing frequency was most important; other important attributes included side effects, changes to the vaginal environment, and changes in menstrual bleeding, although the importance of those attributes differed by country (19). For the majority of couples, either both members had similar individual preferences or, where individual preferences differed, there was equal decision-making around MPT preferences during a joint couple DCE (29). The TRIO study randomized women to a placebo MPT delivery form (injection, tablet, and ring) for one month for each form, and then allowed participants to choose one of these products for an additional two months. During the choice period, the majority chose injection, with no difference in the percentage choosing tablet and ring (30). In addition, mean ratings for how much one liked using the product increased after use (31).

While these findings may help product developers better understand end-user preferences, there are still several socio-behavioral research gaps that may help inform the path forward for MPT development. First, partners have been infrequently included in MPT preference research. Although for some users MPT choice may be an independent decision, with a preference for products that can be used discreetly without partner knowledge, for other users MPT use and preferences may be decided on as a couple. Understanding which users make decisions about MPTs on their own vs. as a couple, and how these decisions are jointly made, is an important research gap. Second, although there are data on MPT preferences, additional research is needed to better understand whether preferences differ over time and across subgroups of women. For example, preferences may differ across adolescents vs. older women of reproductive age, women in long-term relationships vs. those not in a relationship, during breastfeeding, or those in rural vs. urban areas, where easy access to a health facility and product storage may be important considerations. Developmental, social, and cultural considerations may also influence product preferences and should be addressed, particularly given concerns around community acceptability (26) and differing preference findings by country (19, 30). Another aspect of MPT preference not well studied is how potential differential rates of effectiveness for HIV/STI prevention and contraception may impact product preferences. Providers are another type of MPT end user, and their preferences in prescribing MPTs over separate methods is not well-studied.

Research could also benefit from better integration of end user preferences as products are being developed. This integration of behavioral research alongside product development may be beneficial because it: (a) may help product developers better target resources towards developing products that people want and will ultimately use and persist on; and (b) allows for easier product modifications, as even minor changes to a product can be expensive and difficult to make once a product has received regulatory approval. Where possible, it may be important to allow user testing of prototypes, as perceptions of a product may change once a product is actually used (31). For example, the USAID-funded MATRIX project is using an innovative “Design to Delivery (D2D)^7^” approach to integrate end user and key influencer feedback into early stages of product development in an iterative way to inform product-related decisions.

### Uptake, adherence, and persistence on MPTs

Even if an MPT is highly acceptable and includes preferred product attributes, it will be important to study whether there is indeed improved uptake, adherence and persistence over that for separate products (32). MPT adherence, the extent to which the product is used as intended, encompasses an understanding of timing of MPT use, dosage, consistency and duration of use (33). Another factor impacting adherence may be the level of discreteness or confidentiality an MPT affords over separate methods. Further, an MPT that includes a contraceptive purpose may help to reduce stigma associated with products that prevent only HIV/STIs, potentially additionally improving product uptake and adherence relative to HIV/STI prevention products. Interventions and/or tools to address adherence facilitators and barriers will also need to be built into research studies to optimize the effectiveness of MPTs. Such approaches could also be coupled with qualitative research to understand what product characteristics women, couples and providers like and dislike over time, as well as how to best discuss changing needs, to be informative for future generations of MPT product development.

## Implementation science gaps

### MPT implementation

Once an MPT has been developed and found to be effective, implementation science can help clinicians, program managers, and policy makers best understand how to implement the MPT to maximize product delivery and use. First, researchers can identify how to best help patients make informed choices about whether an MPT fits with their values, goals, and lifestyles, for example, through the development of shared decision-making tools (34) that can be used to guide patient-provider discussions. Once multiple MPTs are available, these shared decision-making tools can be expanded to help patients decide which, if any, MPT is the best option for them.

A key area for implementation science research will be to identify barriers and facilitators to implementation of an MPT and to develop implementation strategies that are feasible and effective. Identification of barriers and facilitators to implementation can be investigated through qualitative interviews or focus groups with end-users, including patients, providers, and policy makers. Some of this work can begin even without an available MPT; for example, interviews around barriers and facilitators can be conducted during clinical effectiveness trials. One major challenge for implementation will be to identify the best setting(s) in which to deliver MPTs, particularly for MPTs that address both pregnancy and STI/HIV prevention, as family planning providers may not be comfortable prescribing HIV prevention, and STI/HIV providers may not be comfortable prescribing contraception. Research is needed to understand barriers to product switching and product distribution in different settings; for example, STI/HIV providers may be willing to prescribe an MPT but may not be comfortable prescribing contraception only if someone wants to switch to a single use contraceptive product. Implementation science can help to understand the barriers to MPT delivery, can devise and test strategies to overcome these barriers, and can work with patients and providers to understand their preferences for MPT delivery settings, to ultimately inform the delivery of MPTs when available. Timely implementation research is important in the early stages of product roll out, to inform subsequent larger scale-up.

## Discussion

MPTs hold strong promise for preventing HIV, other STIs, and pregnancy. However, several research gaps must be addressed to ensure MPTs can realize this potential. The development of combination products for multiple therapeutic indications has trailed behind other products as researchers grapple with co-formulating multiple drugs into innovative dosage forms while addressing potential drug-drug interactions and manufacturing challenges. Yet, the potential public health impact of overcoming these issues with innovative solutions is immense. Because blanket regulatory guidance does not exist, researchers and developers could consider the following steps: communicate with regulatory bodies, read regulatory guidance, think globally, consider the entire development pathway as early as possible, seek partners and collaborators, prepare for change, know the market, and communicate with the field. End-users have indicated a strong preference for prevention technologies with multiple indications. However, additional research on end-user preferences integrated into early product development is needed, including research focused on individuals, partners, and providers. Research is needed to understand MPT uptake, adherence, and persistence, as well as social factors that may impact these outcomes. Finally, implementation research is needed to understand barriers to implementation and to test strategies to overcome those barriers.

## Footnotes

^1^ https://www.who.int/news-room/fact-sheets/detail/prequalification-of-medicines-by-who

^2^ https://www.accessdata.fda.gov/scripts/cdrh/cfdocs/cfcfr/cfrsearch.cfm?fr=3.2

^3^ https://www.fda.gov/regulatory-information/search-fda-guidance-documents/principles-premarket-pathways-combination-products

^4^ https://www.ema.europa.eu/en/clinical-development-fixed-combination-medicinal-products-scientific-guideline

^5^ https://www.ecfr.gov/current/title-21/chapter-I/subchapter-A/part-3/subpart-A/section-3.4

^6^ https://theimpt.org/mpt-regulatory-pathways-case-studies-from-mpt-product-developers/

^7^ https://www.matrix4prevention.org/activity-hubs/design-delivery-d2d
